# Mouse mutant phenotyping at scale reveals novel genes controlling bone mineral density

**DOI:** 10.1371/journal.pgen.1009190

**Published:** 2020-12-28

**Authors:** Anna L. Swan, Christine Schütt, Jan Rozman, Maria del Mar Muñiz Moreno, Stefan Brandmaier, Michelle Simon, Stefanie Leuchtenberger, Mark Griffiths, Robert Brommage, Piia Keskivali-Bond, Harald Grallert, Thomas Werner, Raffaele Teperino, Lore Becker, Gregor Miller, Ala Moshiri, John R. Seavitt, Derek D. Cissell, Terrence F. Meehan, Elif F. Acar, Christopher J. Lelliott, Ann M. Flenniken, Marie-France Champy, Tania Sorg, Abdel Ayadi, Robert E. Braun, Heather Cater, Mary E. Dickinson, Paul Flicek, Juan Gallegos, Elena J. Ghirardello, Jason D. Heaney, Sylvie Jacquot, Connor Lally, John G. Logan, Lydia Teboul, Jeremy Mason, Nadine Spielmann, Colin McKerlie, Stephen A. Murray, Lauryl M. J. Nutter, Kristian F. Odfalk, Helen Parkinson, Jan Prochazka, Corey L. Reynolds, Mohammed Selloum, Frantisek Spoutil, Karen L. Svenson, Taylor S. Vales, Sara E. Wells, Jacqueline K. White, Radislav Sedlacek, Wolfgang Wurst, K. C. Kent Lloyd, Peter I. Croucher, Helmut Fuchs, Graham R. Williams, J. H. Duncan Bassett, Valerie Gailus-Durner, Yann Herault, Ann-Marie Mallon, Steve D. M. Brown, Philipp Mayer-Kuckuk, Martin Hrabe de Angelis, IMPC Consortium

**Affiliations:** 1 MRC Harwell Institute, Mammalian Genetics Unit, Harwell Campus, Oxfordshire, United Kingdom; 2 German Mouse Clinic, Institute of Experimental Genetics, Helmholtz Zentrum München, German Research Center for Environmental Health GmbH, Neuherberg, Germany; 3 German Center for Diabetes Research (DZD), Neuherberg, Germany; 4 Czech Center for Phenogenomics, Institute of Molecular Genetics of the Czech Academy of Sciences,Vestec, Czech Republic; 5 Université de Strasbourg, CNRS, INSERM, IGBMC, Illkirch, France; 6 Research Unit of Molecular Epidemiology, Institute of Epidemiology, Helmholtz Zentrum München, Neuherberg, Germany; 7 Mouse Informatics Group, Wellcome Sanger Institute, Hinxton, United Kingdom; 8 Internal Medicine Nephrology and Center for Computational Medicine & Bioinformatics, University of Michigan, Ann Arbor, Michigan, United States of America; 9 Institute of Experimental Genetics, Helmholtz Zentrum München, German Research Center for Environmental Health GmbH, Neuherberg, Germany; 10 University of California-Davis School of Medicine, Sacramento, California, United States of America; 11 Molecular and Human Genetics, Baylor College of Medicine, Houston, Texas, United States of America; 12 Department of Surgical & Radiological Sciences, University of California, Davis, California, United States of America; 13 European Molecular Biology Laboratory- European Bioinformatics Institute, Wellcome Genome Campus, Hinxton, United Kingdom; 14 The Center for Phenogenomics, Toronto, Ontario, Canada; 15 The Hospital for Sick Children, University of Toronto, Toronto, Ontario, Canada; 16 Department of Statistics, University of Manitoba, Winnipeg, Manitoba, Canada; 17 Mouse Pipelines, Wellcome Sanger Institute, Hinxton, United Kingdom; 18 Lunenfeld-Tanenbaum Research Institute, Sinai Health System, Toronto, Ontario, Canada; 19 Université de Strasbourg, CNRS, INSERM, IGBMC, PHENOMIN-ICS, Illkirch, France; 20 The Jackson Laboratory, 600 Main Street, Bar Harbor, Maine, United States of America; 21 MRC Harwell Institute, Mary Lyon Centre, Harwell Campus, Oxfordshire, United Kingdom; 22 Departments of Molecular Physiology & Biophysics, Baylor College of Medicine, One Baylor Plaza, Houston,Texas, United States of America; 23 Dan L Duncan Comprehensive Cancer Center, Baylor College of Medicine, One Baylor Plaza, Houston, Texas, United States of America; 24 Molecular Endocrinology Laboratory, Department of Metabolism, Digestion and Reproduction, Imperial College London, Hammersmith Campus, London, United Kingdom; 25 Advanced Technologies Cores, Baylor College of Medicine, One Baylor Plaza, Houston Texas, United States of America; 26 Institute of Developmental Genetics, Helmholtz Zentrum München, German Research Center for Environmental Health GmbH, Neuherberg, Germany; 27 Chair of Developmental Genetics, TUM School of Life Sciences (SoLS), Technische Universität München, Freising, Germany; 28 Deutsches Institut für Neurodegenerative Erkrankungen (DZNE) Site Munich, Munich, Germany; 29 Munich Cluster for Systems Neurology (SyNergy), Adolf-Butenandt-Institut, Ludwig-Maximilians-Universität München, Munich, Germany; 30 Department of Surgery, School of Medicine and Mouse Biology Program, University of California Davis; 31 Garvan Institute of Medical Research, Sydney, New South Wales, Australia; 32 St Vincent’s Clinical School, Faculty of Medicine, Sydney, New South Wales, Australia; 33 School of Biotechnology and Biomolecular Sciences, UNSW Australia, Sydney, New South Wales, Australia; 34 Chair of Experimental Genetics, TUM School of Life Sciences (SoLS), Technische Universität München, Freising, Germany; Max Planck Institute for Molecular Genetics, GERMANY

## Abstract

The genetic landscape of diseases associated with changes in bone mineral density (BMD), such as osteoporosis, is only partially understood. Here, we explored data from 3,823 mutant mouse strains for BMD, a measure that is frequently altered in a range of bone pathologies, including osteoporosis. A total of 200 genes were found to significantly affect BMD. This pool of BMD genes comprised 141 genes with previously unknown functions in bone biology and was complementary to pools derived from recent human studies. Nineteen of the 141 genes also caused skeletal abnormalities. Examination of the BMD genes in osteoclasts and osteoblasts underscored BMD pathways, including vesicle transport, in these cells and together with *in silico* bone turnover studies resulted in the prioritization of candidate genes for further investigation. Overall, the results add novel pathophysiological and molecular insight into bone health and disease.

## Author summary

Patients affected by osteoporosis frequently present with decreased BMD and increased fracture risk. Genes are known to control the onset and progression of bone diseases such as osteoporosis. Therefore, we aimed to identify osteoporosis-related genes using BMD measures obtained from a large pool of mutant mice genetically modified for deletion of individual genes (knockout mice). In a collaborative endeavor involving several research sites world-wide, we generated and phenotyped 3,823 knockout mice and identified 200 genes which regulated BMD. Of the 200 BMD genes, 141 genes were previously not known to affect BMD. The discovery and study of novel BMD genes will help to better understand the causes and therapeutic options for patients with low BMD. In the long run, this will improve the clinical management of osteoporosis.

## Introduction

Osteoporosis is a common disease, characterized by decreased BMD and increased fracture risk, which causes a profound health burden world-wide [1]. With the discovery that BMD traits are heritable [2], the interest in identifying associated genes has led to a series of studies [3–7]. However, only a limited number of osteoporosis-related genes have already been discovered [8], prompting continuing interest in genes controlling BMD. More recently, three sophisticated genome-wide association studies (GWAS) on osteoporosis and osteoarthritis genes were reported [9–11]. They demonstrated the power of GWAS for the rapid identification of new human genetic loci likely to be associated with osteoporosis. However, the identification of the specific genes that cause BMD alterations remained challenging. Both aforementioned GWAS analyses on osteoporosis relied on the assessment of mouse knockout models to corroborate the proposed candidate genes [9, 10]. This stressed the value of knockout mouse models for the true identification of genes controlling BMD.

**Fig 1:**
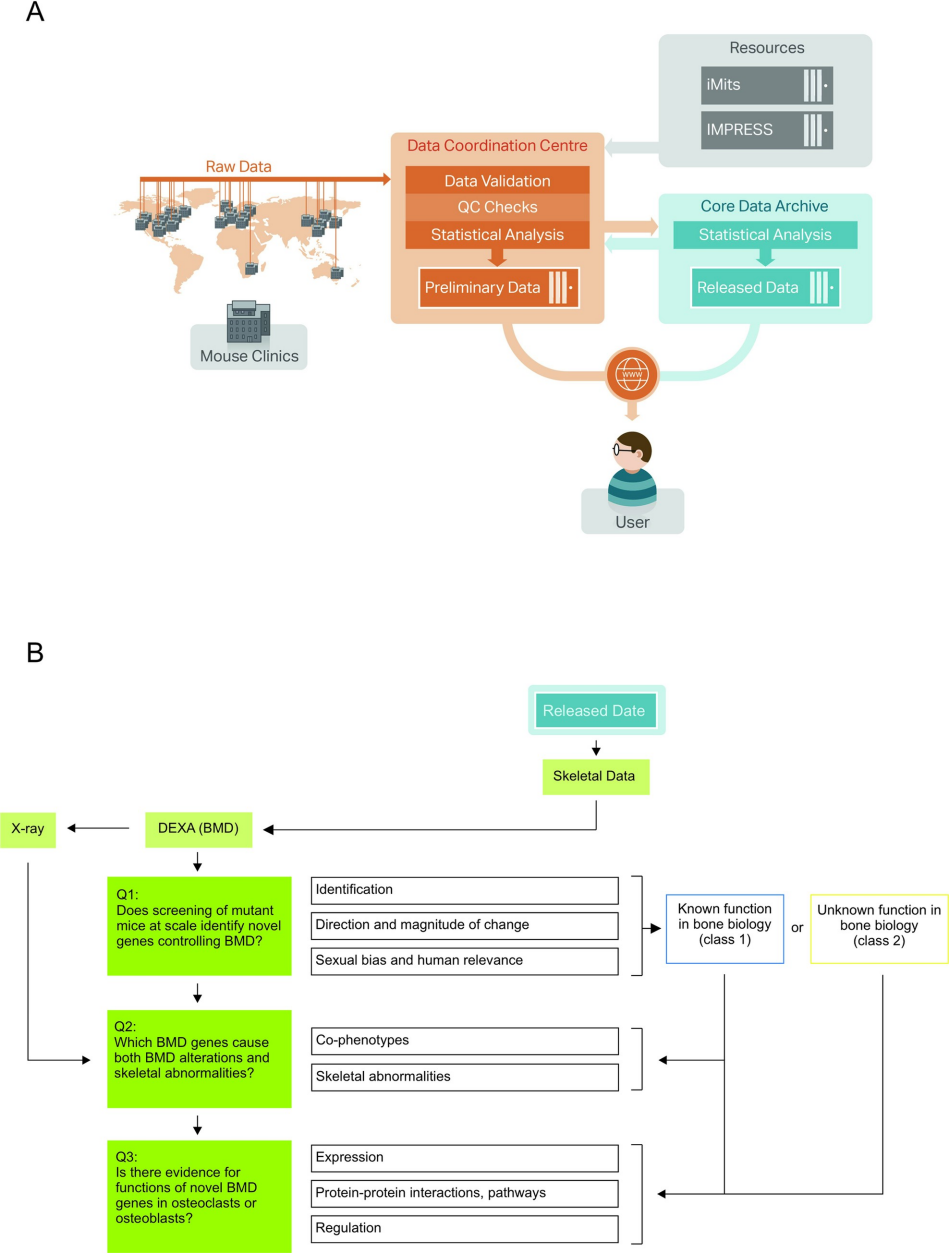
IMPC work-flow and study design. (A) Standardized and automated data flow enabled the large-scale mouse phenotyping under the IMPC program. Shown is the flow of data from IMPC phenotyping mouse clinics to the IMPC Data Coordination Centre for validation, quality control (QC) and preliminary analysis. Work at the Data Coordination Center is governed by uniform operating procedures detailed in IMPReSS (International Mouse Phenotyping Resource of Standardized Screens), while iMits (International Micro-Injection Tracking System) enables efficient and coordinated mice strain production. The Core Data Archive provides final analysis, prior to data release. Standardization, data quality control, an automated statistical analysis pipeline, and the phenotyping of reference strains to assess inter-center variation all help ensure robust and reproducible data. (B) The IMPC skeletal data was investigated. It encompassed primarily BMD measures but also a X-ray-based detection of skeletal abnormalities. Our investigation was guided by three successive research questions. BMD genes with known and unknown function in bone biology were categorized as class 1 (blue) and class 2 (yellow) BMD genes, respectively.

Beyond the assessment of individual knockout mice for data corroboration, a more systematic and comprehensive production and skeletal phenotyping of knockout mice has been part of the IMPC, an international consortium of mouse clinics (Fig 1A) [12–14]. Work at the IMPC aims to individually ablate the function of every protein-coding gene in mice, and subsequently phenotype the mutated animals [15–18]. The IMPC phenotyping pipeline interrogates a range of organ and tissue types, including the skeleton. The skeletal exams encompass dual-energy X-ray absorptiometry (DXA)-based measures of the three important bone parameters bone area (BA), bone mineral content (BMC), and the resulting BMD. Further to this, planar radiographic images are used for detection of skeletal abnormalities. This study probed the IMPC data release 6.0 and focused on genes controlling BMD. This measure was selected because in the clinical setting, in veterinary medicine, and in genetic mouse models low BMD is a hallmark of osteoporosis [19–21]. The present study has been driven by the following three research questions: (1) does screening of mutant mice at scale identify novel genes controlling BMD?, (2) which BMD genes cause both BMD alterations and skeletal abnormalities?, and (3) is there evidence for functions of novel BMD genes in osteoblasts or osteoclasts? (Fig 1B). Addressing these questions, we report on a total of 200 BMD genes that, when deleted, caused alteration in BMD. Of those 200 genes, 59 loci (class 1) had a previously reported function in bone, while 141 (class 2) genes were not previously described as regulators of BMD in mouse or human. Using a combination of IMPC phenotyping data with traditional bioinformatics and an innovative *in silico* bone turnover model, enabled insight into the biology of BMD maintenance and identified novel skeletal candidate genes, including *Arl4d*, *Ncald*, and *Rab3ip*, for further investigation.

## Results

### Identification and characterization of BMD Genes

**Fig 2:**
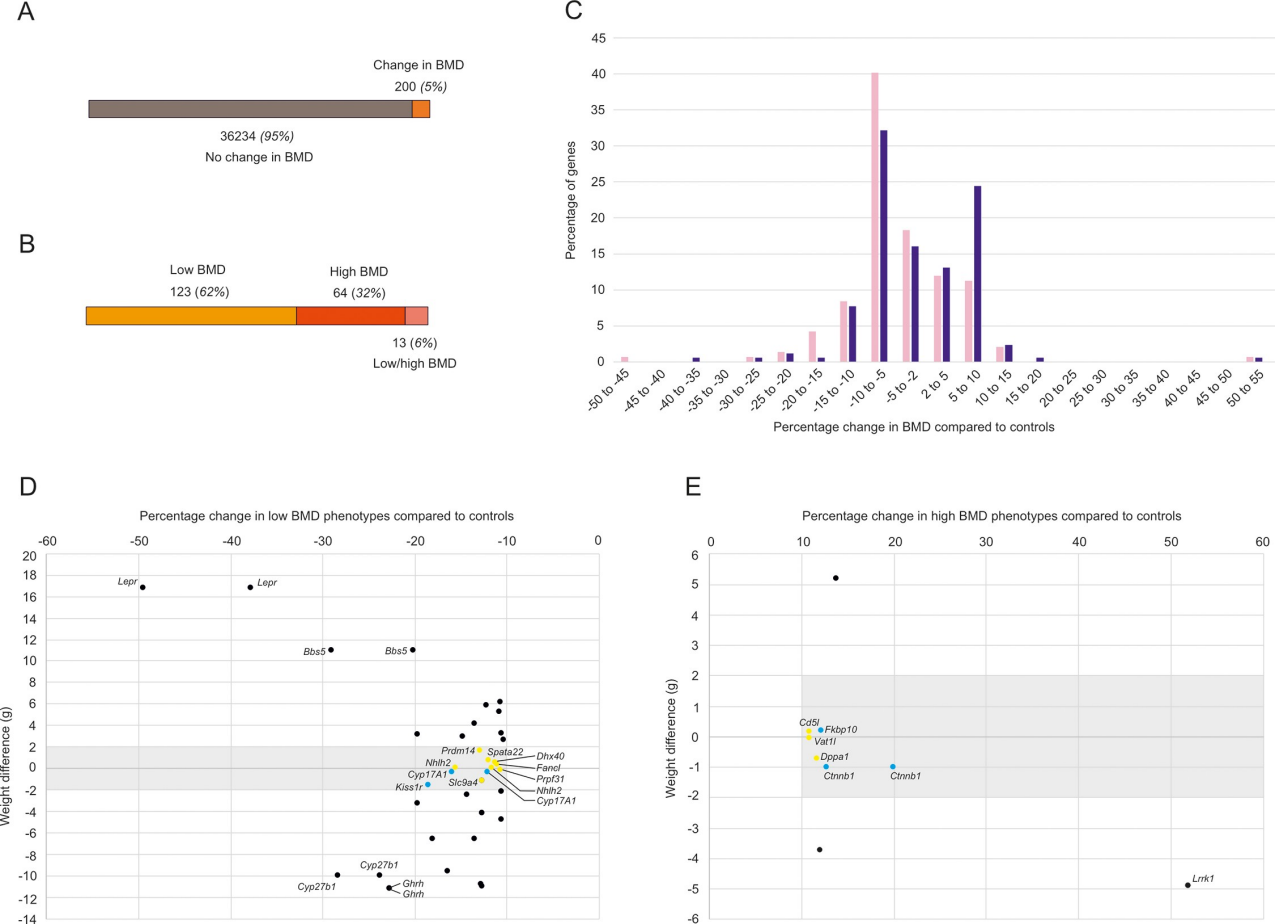
Mouse phenotyping identified 200 BMD genes. (A) Of the 3,823 genes deleted in mice, 200 or 5% caused a change in BMD phenotype. (B) Directional changes in BMD. Low and high BMD was found after deletion of 123 and 64 genes, respectively. Another 13 genes caused low or high BMD dependent on sex. (C) Percentage change in BMD compared to wt animals. In both male (blue bars) and female (pink bars) mice, most BMD genes reduced BMD between 5% and 10%. Changes between -2% and +2% were not plotted as these were not considered by PhenStat as significant phenotypes. (D) and (E) A limited weight difference compared to wt controls in combination with a greater than 10% decrease or increase in BMD change prioritized (gray shaded areas) genes for further investigation. Genes with a greater than 10% decrease (D) or increase (E) in BMD were plotted separately, and genes affecting female or male mice represented individually. Priority genes in the gray shaded areas were colored yellow or blue according to class 1 or 2, respectively. For clarity, outside the gray sectors, only genes associated with greater than 20% decrease (D) or increases (E) in BMD were name tagged.

Bone mineral density data on 3,823 genes were recorded and analyzed (Fig 1A). Within this gene set, we identified 200 (5%) genes that caused a statistically significant (p<0.0001) decrease or increase in BMD when compared to wt control animals (Fig 2A and S1 and S2 Tables. Together, the pool of 200 BMD genes was then analyzed further. A majority, 123 genes, produced a low BMD phenotype, while 64 genes caused a high BMD phenotype (Fig 2B). In addition, 13 genes were associated with either a low or high BMD phenotype, depending on sex (Figs 2B and S1). Thus, the 200 BMD genes were derived from 213 mutant mouse lines, of which 161, 47, and 5 were homozygotes, heterozygotes, and hemizygotes, respectively (S1 Table). The identity of the 200 BMD genes is shown in Table 1 and S1 Table. With the exception of 4930591A17Rik, biological functions of all BMD genes had been previously reported to some extent. To determine if novel BMD loci were among the 200 genes, we discriminated between genes with a known (class 1) and unknown (class 2) function in bone biology based on previously published data. A total of 59 and 141 genes were assigned to classes 1 (blue) and 2 (yellow), respectively (Table 1).

**Table 1:** Identity and classification of the 200 genes controlling BMD.

*Acp2* (ACP2)	*Ahrr* (AHRR)	*Aktip* (AKTIP)	*Aldh2* (ALDH2)	*Alg13* (ALG13)	*Angptl4* (ANGPTL4)	*Ankrd11* (ANKRD11)
*Atp11a* (ATP11A)	*Bbs5* (BBS5)	*Bhlhe40* (BHLHE40)	*Brpf1* (BRPF1)	*Chd9* (CHD9)	*Ckb* (CKB)	*Col1a2* (COL1A2)
*Ctnnb1* (CTNNB1)	*Ctsk* (CTSK)	*Cyp17a1* (CYP17A1)	*Cyp27b1* (CYP27B1)	*Dnmt3a*	*Duoxa2* (DUOXA2)	*Fkbp10* (FKBP10)
				(DNMT3A)		
*Foxo3* (FOXO3)	*Ghrh* (GHRH)	*Ghrhr* (GHRHR)	*Hdac8* (HDAC8)	*Hgsnat* (HGSNAT)	*Il6st* (IL6ST)	*Iqgap1* (IQGAP1)
*Itch* (ITCH)	*Kiss1r* (KISS1R)	*Kmt2a* (KMT2A)	*Lepr* (LEPR)	*Lrrk1* (LRRK1)	*Macrod2* (MACROD)	*Map3k1* (MAP3K1)
*Miga2* (MIGA2)	*Mysm1* (MYSM1)	*Ndrg1* (NDRG1)	*P3h1* (P3H1)	*Pank3*	*Pclaf* (PCLAF)	*Plcg2* (PLCG2)
				(PANK3)		
*Plekhm1* (PLEKHM1)	*Plod1* (PLOD1)	*Pls3* (PLS3)	*Pstpip2* (PSTPIP29	*Rbpj* (RBPJ)	*Satb1* (SATB1)	*Sms* (SMS)
*Sparc* (SPARC)	*Spns2* (SPNS2)	*Stx16* (STX16)	*Tcf4* (TCF4)	*Thra* (THRA)	*Tnfrsf9* (TNFRSF9)	*Tram2* (TRAM2)
*Trim37* (TRIM37)	*Txnip* (TXNIP)	*Wnt10b* (WNT10B)	*1700008O03Rik* (C19orf81)	*2210408I21Rik* (KIAA0825)	*2610318N02Rik* (N/A)	*A730017C20Rik* (KIAA1024L)
*Abcb11* (ABCB11)	*Acmsd* (ACMSD)	*Acsf2* (ACSF2)	*Adam32* (ADAM32)	*Adgrd1* (ADGRD1)	*Adgrf5* (ADGRF5)	*Adnp2* (ADNP2)
*Adpgk* (ADPGK)	*Aldh3b1* (ALDH3B1)	*Aldob* (ALDOB)	*Ang6* (ANG)	*Ap4e1* (AP4E1)	*Aptx* (APTX)	*Atg4a* (ATG4A)
*Arhgef4* (ARHGEF4)	*Arl4d* (ARL4D)	*Atp8a1* (ATP8A1)	*Atxn10*	*Babam2* (BABAM2)	*Bach2* (BACH2)	*Bbx* (BBX)
			(ATXN10)			
*BC030499* (SGK494)	*BC055324* (C1orf112)	*Cd5l* (CD5L)	*Chsy3* (CHSY3)	*Clcf1* (CLCF1)	*Clpp* (CLPP)	*Clvs1* (CLVS1)
*Colca2* (COLCA2)	*Cops4* (COPS4)	*Cpsf3*	*D630023F18Rik* (C2orf80)	*Dagla* (DAGLA)	*Defb14* (DEFB103A)	*Ddhd1* (DDHD1)
		(CPSF3)				
*Dhx40* (DHX40)	*Dnase1l2* (DNASE1L2)	*Dnase2b* (DNASE2B)	*Dok7* (DOK7)	*Dopey2* (DOPEY2)	*Dppa1* (N/A)	*Dscc1* (DSCC1)
*Elk4*	*Entpd6*	*Fahd2a* (FAHD2A)	*Fam160a1* (FAM160A1)	*Fancl* (FANCL)	*Farsa* (FARSA)	*Fbxo38* (FBXO38)
(ELK4)	(ENTPD6)					
*Fndc9* (FNDC9)	*Fntb*	*Frmd7* (FRMD7)	*Fundc1* (FUNDC1)	*Gpr152* (GPR152)	*Gpr182* (GPR182)	*Gtf2a1* (GTF2A1)
	(FNTB)					
*Hbs1l* (HBS1L)	*Hectd3* (HECTD3)	*Hook3* (HOOK3)	*Iqsec3* (IQSEC3)	*Kcnj16* (KCNJ16)	*Mcph1* (MCPH1)	*Mid2* (MID2)
*Mir96* (MIR96)	*Mlec* (MLEC)	*Mmachc* (MMACHC)	*Mogs* (MOGS)	*Myh1* (MYH1)	*Nbeal2* (NBEAL2)	*Ncald* (NCALD)
*Nhlh2* (NHLH2)	*Ogfod3* (OGFOD3)	*Osbpl3* (OSBPL3)	*Parvb*	*Pbx3* (PBX3)	*Pdx1* (PDX1)	Pex3 (PEX3)
			(PARVB)			
*Pfkfb3* (PFKFB3)	*Phf19* (PHF19)	*Pkp4*	*Pld5* (PLD5)	*Ppp3r2* (PPP3R2)	*Pptc7* (PPTC7)	*Prdm14* (PRDM14)
		(PKP4)				
*Prpf31* (PRPF31)	*Prss2* (PRSS2)	*Pskh1* (PSKH1)	*Ptafr* (PTAFR)	*Pxylp1* (PXYLP1)	*Rab11fip3* (RAB11FIP3)	*Rab3ip* (RAB3IP)
*Rbm25* (RBM25)	*Rbsn* (RBSN)	*Rnf169* (RNF169)	*Rsf1* (RSF1)	*Sdsl* (SDSL)	*Serf1* (SERF1A)	*Serinc3* (SERINC3)
*Setd1a* (SETD1A)	*Sh3gl2* (SH3GL2)	*Sirt2* (SIRT2)	*Slc4a10* (SLC4A10)	*Slc5a5* (SLC5A5)	*Slc9a4* (SLC9A4)	*Slc25a30* (SLC25A30)
*Slc38a10* (SLC38A10)	*Slc35f6* (SLC35F6)	*Slx4* (SLX4)	*Snap29* (SNAP29)	*Spata22* (SPATA22)	*Spidr* (SPIDR)	*Spin1* (SPIN1)
*Stag3* (STAG3)	*Stard5* (STARD5)	*Stx8* (STX8)	*Syn3* (SYN3)	*Tatdn3* (TATDN3)	*Tmem189* (TMEM189)	*Tmem42* (TMEM42)
*Tnfaip1* (TNFAIP1)	*Tox* (TOX)	*Trh* (TRH)	*Trim39*	*Trip13* (TRIP13)	*Ttc28* (TTC28)	*Ttll4*
			(TRIM39)			(TTLL4)
*Ube2d3* (UBE2D3)	*Ube2j1* (UBE2J1)	*Ube3c* (UBE3C)	*Uhrf2* (UHRF2)	*Vat1l*	*Xk* (XK)	*Xylb* (XYLB)
				(VAT1L)		
*Zbtb45* (ZBTB45)	*Zfp36* (ZFP36)	*Zfp704*	*4930591A17Rik* (N/A)			
		(ZNF704)				

Mouse genes and in parenthesis the human orthologues are presented.

Color: Blue, class 1 genes (known function in bone biology); yellow class 2 genes (unknown function in bone biology): orange (no known function). Shading: Light blue/yellow, decreased BMD; dark blue/yellow, increased BMD; mixed dark and light, decreased/increased BMD dependent on sex. Abbreviation: N/A, not available.

To quantitatively assess the change in BMD caused by the 200 genes, we plotted gene numbers against percentage of change in BMD (Fig 2C). While decreases in BMD ranged from 2% to 49%, and increases from 2% to 52%, we observed that the majority of genes elicited a reduction in BMD between 5% and 10%. PhenStat, the statistical package used for the analysis of the high-throughput phenotype data, did not consider BMD changes between -2% and +2% to be significant phenotypes. A comparison between male and female animals showed an asymmetrical data distribution with about twice as many genes causing a 5% to 10% increased BMD in males than in females (Fig 2C). Regardless of sex, BMD decreased greater than 20% only upon deletion for the class 1 genes *Lepr*, *Bbs5*, *Cyp27b1*, and *Ghrh*, while it increased by more than 20% only after loss of the class 1 gene Lrrk1 (Fig 2D and 2E and S1 Table). To further prioritize the BMD genes, we set criteria by asking which class 2 gene deletions resulted in a greater than 10% decrease or increase in BMD in conjunction with a limited weight change. Weight changes can confound BMD assessments and we set an arbitrary limit to approximately 10% of the body weight of the animal. The 6 low BMD genes *Dhx40*, *Fancl*, *Nhlh2*, *Prdm14*, *Slc9a4*, and *Spata22* (Fig 2D), and the 3 high BMD genes *Cd5l*, *Dppa1*, and *Vat1l* (Fig 2E) met our criteria. Deletion of Nhlh2 and Dppa1 yielded the most pronounced decrease and increase in BMD, respectively.

To survey for a potential role of the 200 BMD genes in human BMD, GWAS data based on the UK Biobank release was interrogated. We selected the UK Biobank because we believe the size of the data set is an advantage that offsets the difference in BMD acquisition technique, which was based on ultrasound and not the X-ray mean used in this study. A distance range between 0 kilo bases (kB) and 250 kb was chosen. Of the 200 genes, 11, 38, and 52 genes matched the human GWAS derived genes at a 0 kB, 100 kB, and 250 kB distance range, respectively (S3 Table). To take a closer look at genes with potential human relevance, we then utilized the GEFOS data set, which although smaller than the UK Biobank release, utilized X-ray-based BMD acquisition. The six class 2 genes *AP4E1*, *KIAA0825*, *ACSF2*, *PKP4*, *TTC28*, *PHF19* and the class 1 gene *MACROD2* altered BMD in humans (S4 Table). The direction of BMD change matched between mouse and human for the five genes *ACSF2*, *PKP4*, *TTC28*, *PHF19* and *MACROD2*, and as described below the latter was found to be expressed in osteoblasts.

### Sexual dimorphism of the BMD genes

**Fig 3:**
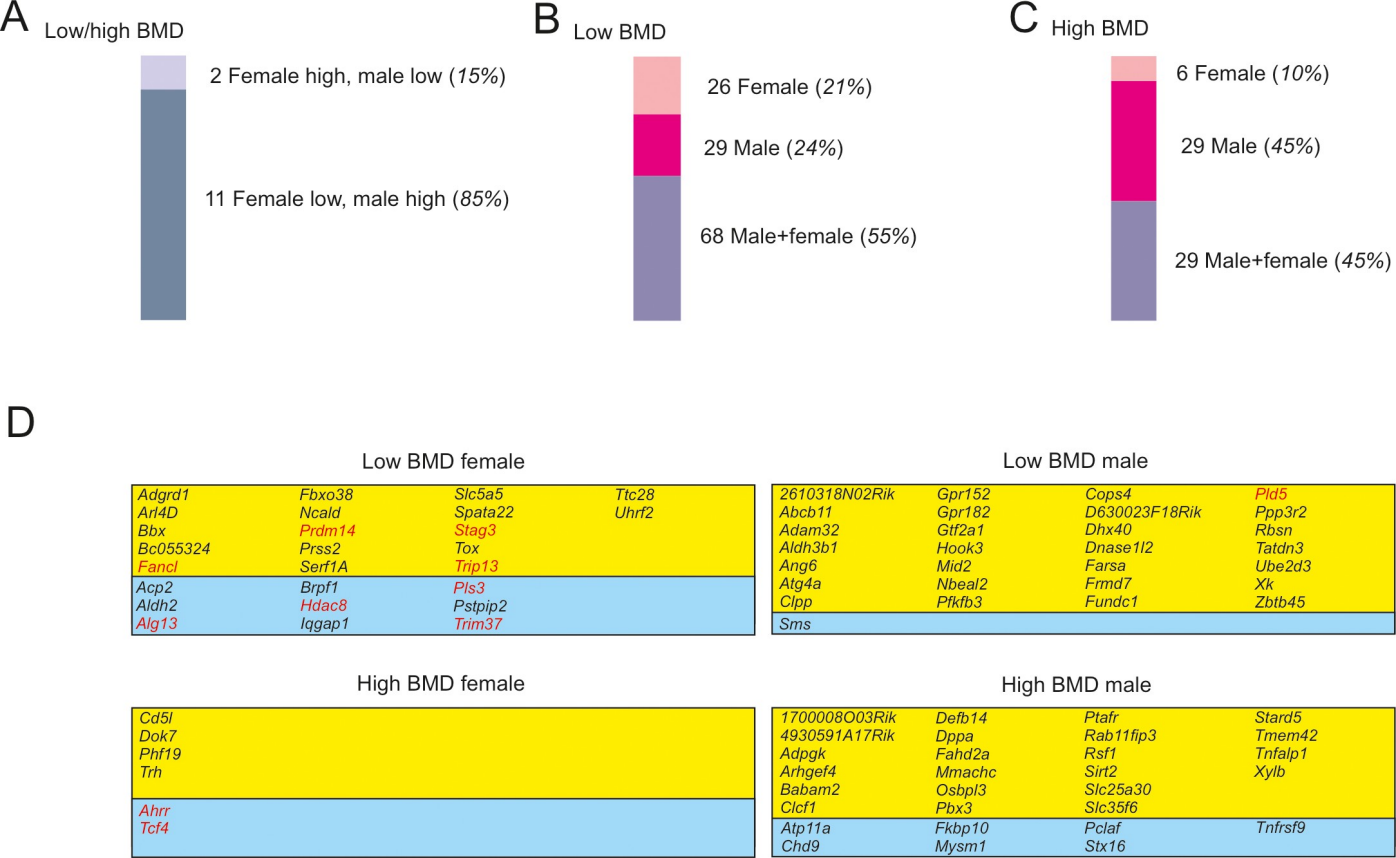
BMD genes displayed sexual dimorphism. (A-C) A subset of 90 genes affected BMD specifically in one sex. The 90 genes encompass in females (pale pink bars) 26 low BMD and 6 high BMD genes plus in males (magenta) 29 low BMD and 29 high BMD genes. (D) The identity of all sexually dimorphic genes grouped by class and direction of BMD change. Genes with a previously reported role in sexual dimorphism other than bone are marked red.

The observation that the direction of BMD change depended on sex in 13 genes prompted further investigation of a potential sexual bias in BMD. Of the 13 genes, 11 genes caused low BMD in females and high BMD in males, and 2 genes produced high BMD in females and low BMD in males (Fig 3A). We then examined genes causing a unidirectional change in BMD. Twenty-six, 29, and 68 genes caused low BMD in female, male and both sexes, respectively (Fig 3B). In comparison, 6, 29, and 29 genes caused high BMD in females, males, and both sexes, respectively (Fig 3C). A cross-comparison independent of the direction of the BMD change, demonstrated that a sub-set of 90 genes regulated BMD exclusively in either females or males. Of those 90 genes, only 11 genes have been previously described in the context of sexual dimorphism in organs other than bone (Fig 3D).

### Pleiotropy of the BMD genes

**Fig 4:**
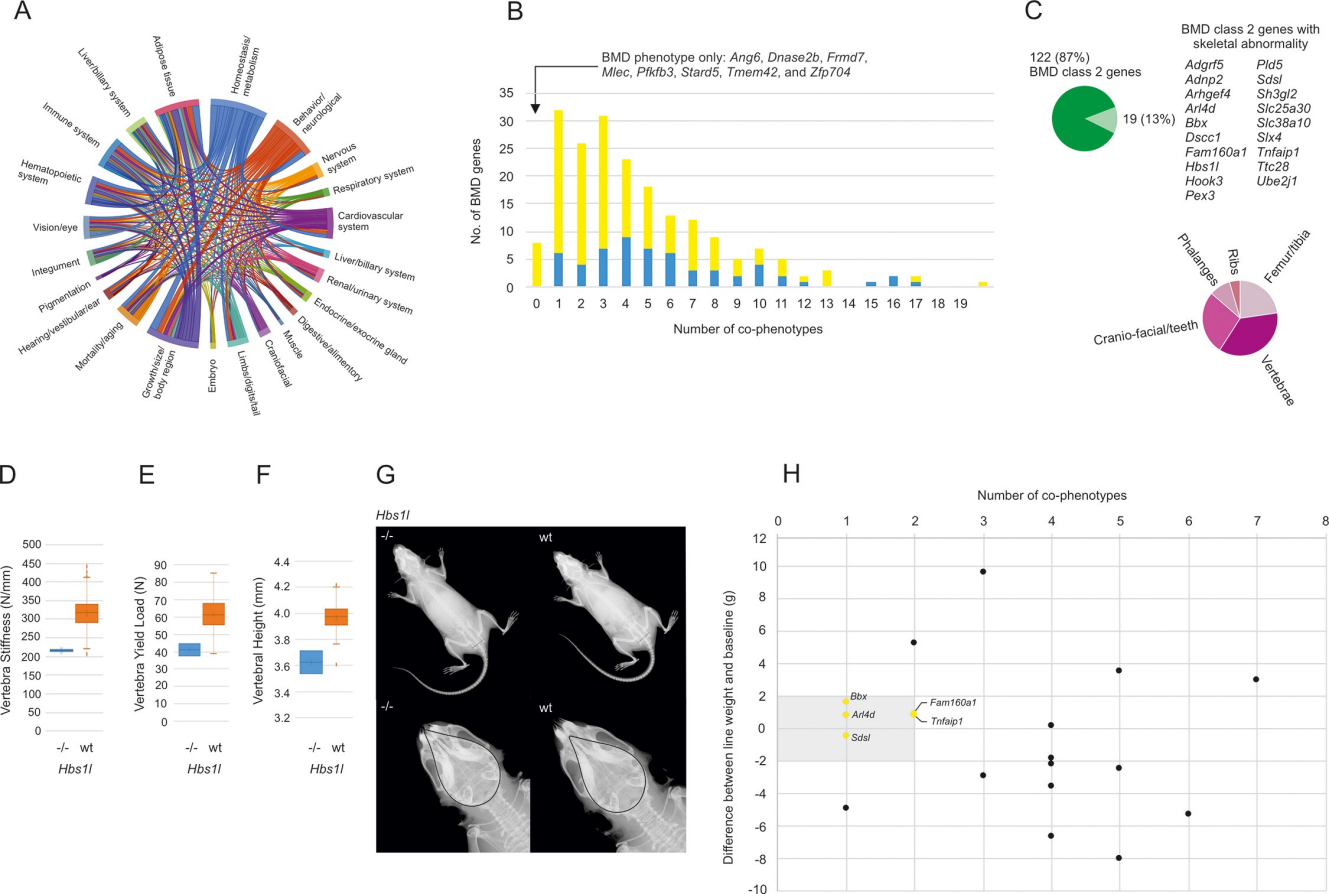
BMD genes were pleiotropic. (A) Chord diagram plotting the inter-connectivity of co-phenotypes associated with the 200 BMD genes. The outer segments represent the co-phenotypes grouped as top-level Mammalian Phenotype (MP) terms. The segment size correlates with the number of genes exhibiting the co-phenotype. Homeostasis/metabolism was the primary co-phenotype detected. The size of the arcs connecting the phenotypes was proportional to the number of genes connected. Diagram colors were assigned clockwise starting with the primary phenotype. (B) The majority of BMD genes presented with up to 3 co-phenotypes, while eight genes were limited to a skeletal phenotype. Class 1 (known function in bone biology) and 2 (unknown function in bone biology) genes are distinguished in yellow and blue color, respectively. (C) Analysis of the IMPC phenotyping data showed that 19 of the 141 class 2 genes were also associated with skeletal abnormalities in addition to changes in BMD. Vertebrae abnormalities were the most frequent skeletal abnormalities associated with the deletion of one of the 19 genes. (D) OBCD data showed a difference in vertebra stiffness for Hbs1l compared to wt. (E) OBCD data detected a difference in vertebra yield load for Hbs1l compared to wt. (F) OBCD data measured a difference in vertebral height for Hbs1l compared to wt. (G) Dorsal images were taken from the IMPC x-ray screen. Representative female *Hbs1l*-deficient mice and wild type (wt) control mice are shown. In the upper panels, the position of the pubic body was denoted by a black line. In *Hbs1l*^-/-^ mice, a distal shift of the pubic body intersection relative to the S1 sacral vertebra was seen (p<0.0001 for vertebrae abnormality in the *Hbs1l*^-/-^ cohort versus wt mice). The two lower panels show a skull close-up on a second set of female animals. Identical outlines of the cranium were superimposed. *Hbs1l*^-/-^ mice showed a compressed cranium including nasal bones (p<1x10^-8^ for vertebrae abnormality in the *Hbs1l*^-/-^ cohort versus wt mice). (H) Among the 19 genes with both BMD change and skeletal abnormality, a limited weight difference compared to wt controls and a low number of co-phenotypes (gray shaded area) indicated genes more likely to directly affect the skeleton. All 19 genes were plotted, genes in the gray shaded areas were colored in yellow according to their class 2 designation.

Taking advantage of the broad phenotyping approach of the IMPC, we screened the 200 BMD genes for additional phenotypes. Alterations in homeostasis/metabolism occurred most frequently (99 genes), followed by changes in growth/size/body region (94 genes), behavior/neurological (92 genes), and adipose tissue (72 genes) (Fig 4A and S5 Table). Next, we determined the inter-relationships of the co-phenotypes. With the exception of expected relationships, for example between homeostasis/metabolism, adipose tissue and growth/size, we observed a mostly uniform distribution of phenotype connections (Fig 4A). In contrast, there was variability in the pleiotropy distribution across the BMD genes. About half of the 200 BMD genes presented with up to 3 co-phenotypes, and only a few genes associated with large numbers of co-phenotypes (Fig 4B). Importantly, no co-phenotypes were detected for the eight class 2 genes *Ang6*, *Dnase2b*, *Frmd7*, *Mlec*, *Pfkfb3*, *Stard5*, *Tmem42*, and *Zfp704*, suggesting they affected the skeleton specifically. To assess if loss of any of these eight genes or any other class 2 genes produced gross skeletal abnormalities in addition to their BMD phenotype, we read the radiographs routinely performed as part of the IMPC phenotyping. None of the eight class 2 genes had both BMD changes and skeletal abnormalities. However, 19 other class 2 genes caused skeletal abnormalities in addition to changes in BMD (Fig 4C). Vertebrae abnormalities were the primary skeletal phenotype associated among the BMD genes (Fig 4C) and were seen in the 9 genes *Adnp2*, *Arhgef4*, *Dscc1*, *Fam160a1*, *Hbs1l*, *Pex3*, *Sh3gl2*, *Slc25a30*, and *Slc38a10*. As a combination of altered BMD and skeletal abnormality has the potential to compromise the biomechanical stability of bone, we explored the biomechanical testing data collected as part of the OBCD project. For this, we facilitated the import of skeletal phenotyping data from the OBCD into IMPC. Then, we surveyed the bones of the 19 knockout mice for compromised mechanical stability, a functional hallmark of osteoporosis. Of the 19 genes with both a BMD and skeletal phenotypes, the three genes *Hbs1l*, *Slc25a30* and *Slc38a10* matched to the OBCD data, but only loss of Hbsl affected skeletal stability, specifically it resulted in a decrease in vertebrae stiffness and yield load (Fig 4D–4F). This demonstrated a functional role of Hsb1l in skeletal maintenance and was consistent with the vertebrae abnormalities detected by radiographic screening (Fig 4G). Interestingly, loss of Hsb1l also caused cranio-facial abnormalities (Fig 4G). With the limited overlap between IMPC and OBCD in mind, we pursued another strategy to isolate good candidate genes. A weight change limited to about 10% and low number of maximal 2 co-phenotypes were used to identify genes more likely to directly affect the skeleton. The five genes *Arl4d*, *Bbx*, *Fam160a1*, *Sdsl*, and *Tnfaip1* fulfilled these two criteria (Fig 4H).

### Function of novel BMD Genes in osteoclasts and osteoblasts

**Fig 5:**
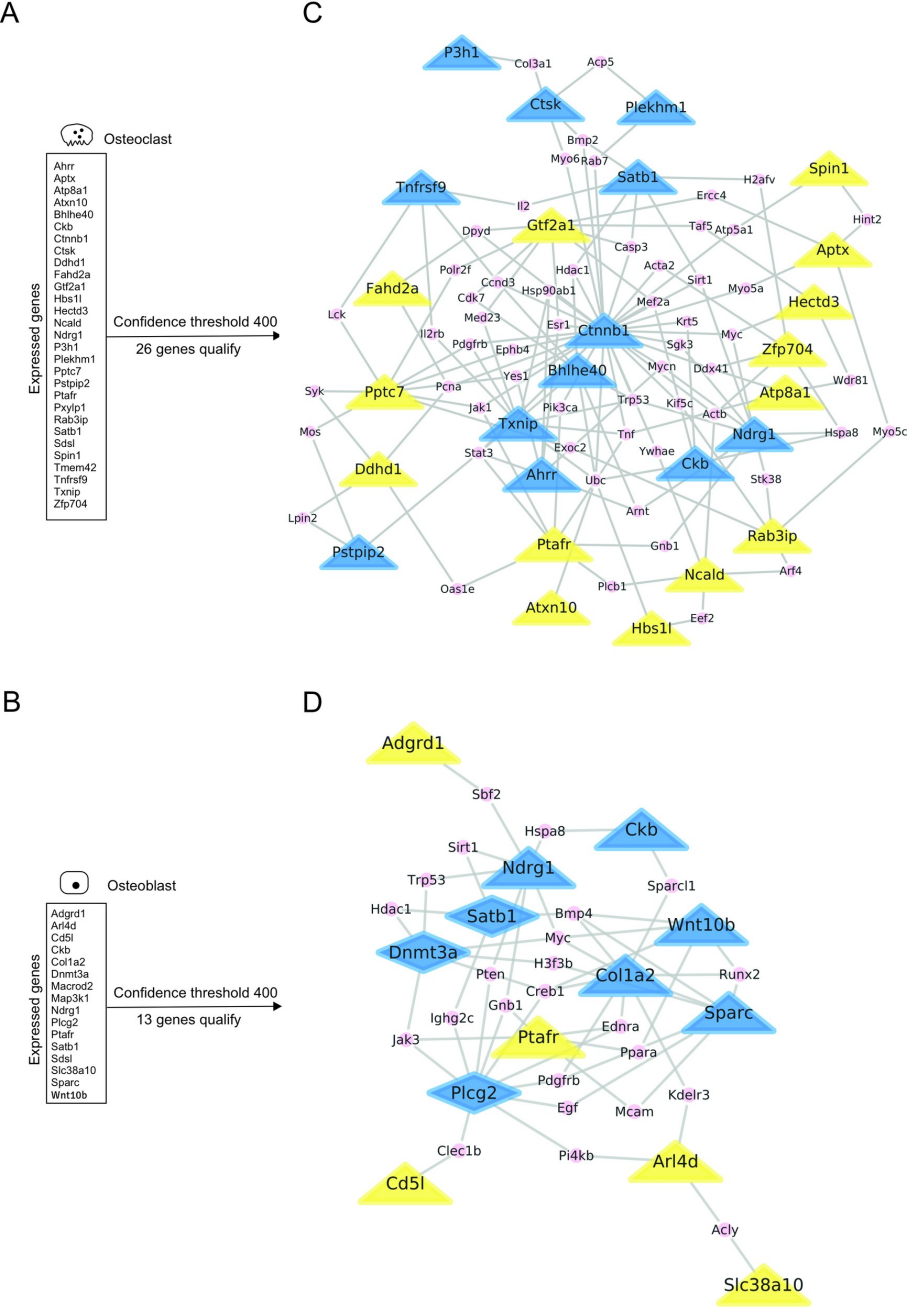
Subsets of BMD proteins mapped specific pathways in osteoclasts and osteoblasts. Using curated NCBI Gene Expression Omnibus data, expression of the 200 BMD genes was mapped in (A) osteoclasts, the bone resorbing cells, and (B) osteoblasts, the bone-forming cells. Protein-protein interactions (PPIs) were identified using the STRING database allowing the annotation of 178 of the 200 BMD genes. The central hubs of the network where identified based on the betweenness centrality index and extracted from the main network. All PPIs were presented as black lines. Blue and yellow nodes represent class 1 (known function in bone biology) and class 2 genes (unknown function in bone biology), respectively. The zygosity of the mutants is indicated by a triangular (homozygous) or diamond (heterozygous) shape of the node. The resulting central protein-protein interactions sub-networks in osteoclasts (C) and osteoblasts (D) are shown. Proteins connecting the BMD gene nodes were represented as small pink nodes.

To offer insight into a potential direct role of the 200 BMD genes in bone we focused on the two principle bone cell types. Using available gene expression data, the 200 genes were assigned to osteoclasts and osteoblasts (Fig 5A and 5B). We found that of the 200 genes 29 and 16 genes were expressed in osteoclasts and osteoblasts, respectively (p<0.05), with an overlap in expression seen for *Cbk*, *Ptafr*, *Satb1*, and *Sdsl*. With respect to the five class 2 genes *Arl4d*, *Bbx*, *Fam160a1*, *Sdsl* and *Tnfaip1* identified above, *Arl4d* and *Sdsl* were expressed in bone cells. Using STRING, we then tested (p<2.2x10^-16^) for protein-protein interactions (PPIs) within the sets of 29 and 16 genes expressed in osteoclasts and osteoblasts, respectively (Fig 5C and 5D). Upon thresholding, 26 osteoclast and 13 osteoblast genes remained for construction of PPI networks. For several reasons we found such networks informative. Firstly, they identified proteins with a large number of interactions and hence hubs that route information. In osteoclasts, the class 1 gene product Ctnnb1 (ß-catenin) formed a central PPI hub (Fig 5C). In addition, the two class 2 genes products Pptc7 and Gtf2a1 served as PPI hubs in these cells. In contrast, in osteoblasts we observed that the class 1 gene product Col1a2 and to a lesser degree the class 1 gene product Plcg2 formed the central PPI hubs (Fig 5D). Secondly, patterns of PPIs uncovered prominent pathways in cells. For example, in osteoclasts we discovered a PPI chain connecting Pptc7, Excoc2, Rab3ip, Arf4, and Ncald (Fig 5C). The latter four proteins, participate in intracellular vesicle trafficking, highlighting the role of this pathway in BMD control via osteoclast function. Thirdly, PPI networks detected previously unknown links in BMD pathways. For instance, in osteoblasts (Fig 5D) the class 2 protein Arl4d interacted with Col1a2 via Kdelr3. Together, these findings support further investigation of the class 2 genes *Pptc7*, *Rab3ip*, *Ncald*, and *Arl4d*, of which the latter three produced a low BMD phenotype upon depletion (Table 1).

**Fig 6:**
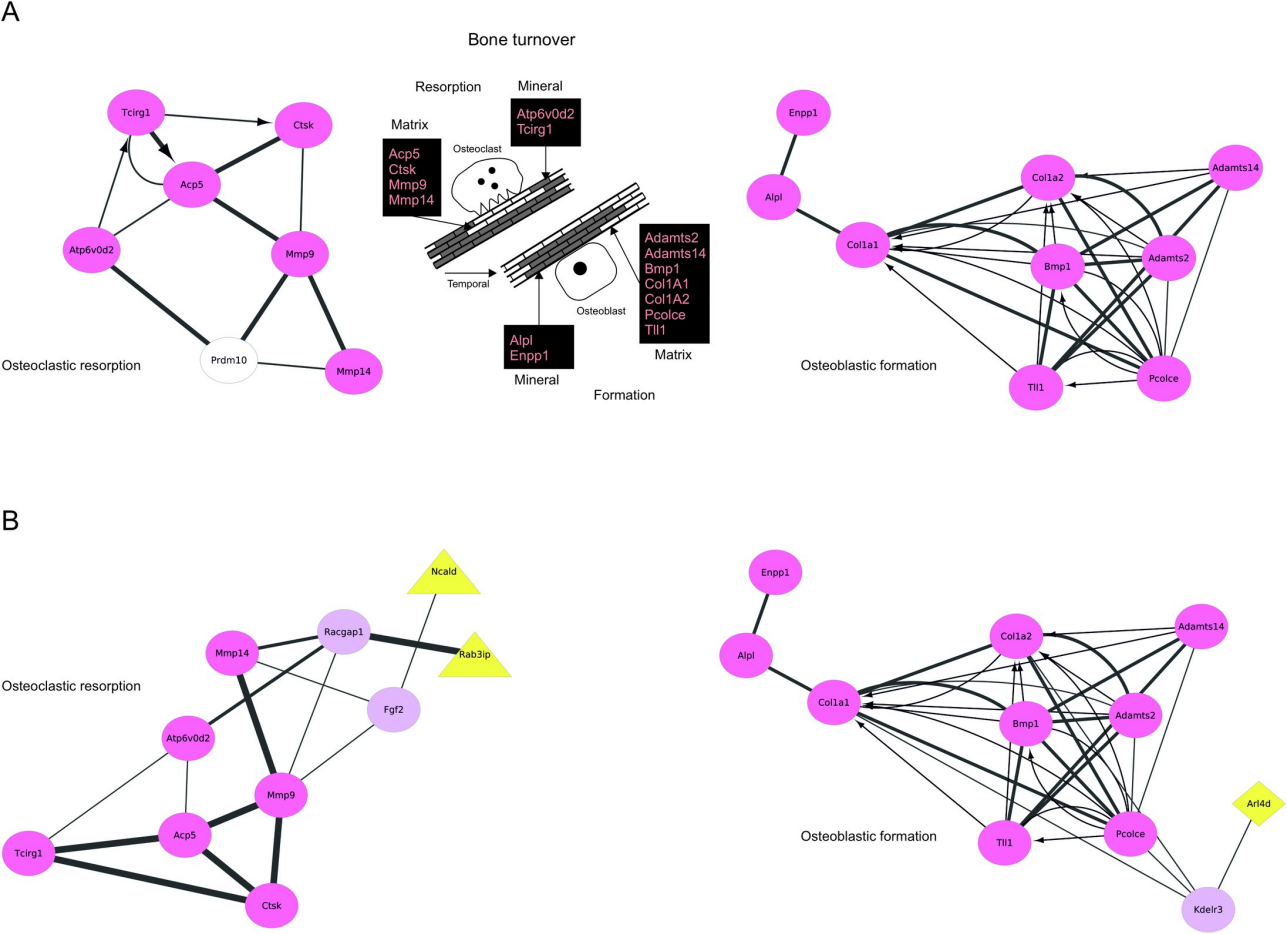
An in silico bone turnover model validated class 2 candidate BMD genes. (A) The *in silico* model of bone turnover captured the extracellular break down of bone and the subsequent formation of new bone. The model is simplified excluding for example many of the intracellular proteins driving bone turnover or non-collagenous matrix proteins. While resorption of both bone matrix and mineral may occur concurrent, bone matrix formation typically precedes mineral deposition. The genes mainly responsible for bone turnover were grouped and annotated as follows. Osteoclastic bone matrix resorption: tartrate-resistant acid phosphatase type 5 (Acp5), essential for osteoclast motility; cathepsin K (Ctsk), matrix break down; matrix metallopeptidase 9, (Mmp9), matrix break down; matrix metallopeptidase 14 (Mmp14), matrix break down. Osteoclastic bone mineral resorption: ATPase H+ transporting V0 subunit D2 (Atp6v0d2), acidic pH and mineral removal; ATPase H+ transporting V0 subunit A3, (Tcirg1), acidic pH and mineral removal. Osteoblastic bone matrix formation: ADAM metallopeptidase with thrombospondin type 1 motif 2. (Adamts2), N-terminal processing of procollagen; ADAM metallopeptidase with thrombospondin type 1 motif 14 (Adamts14), N-terminal processing of procollagen; bone morphogenic protein 1 (Bmp1), C-terminal processing of procollagen; collagen type I alpha 1 chain (Col1a1) and collagen type I alpha 2 chain (Col1a2), procollagen formation; procollagen C-endopeptidase enhancer 1 (Pcolce), enhancer of C-terminal processing of procollagen; tolloid like 1 (Tll1), C-terminal processing of procollagen. Osteoblastic bone mineral deposition: tissue-nonspecific alkaline phosphatase (Alpl), removal of mineralization inhibitor inorganic pyrophosphate (PP_i_); ectonucleotide pyrophosphatase/phosphodiesterase 1 (Enpp1), production of PP_i_. Together, these proteins (red elliptic nodes) formed the PPI networks for osteoclastic resorption and osteoblastic formation during bone turnover. Connecting proteins distinct from the bone turnover genes are represented as pink elliptic nodes, while white nodes marked genes with an undefined role in bone. Black lines represent PPIs as determined by STRING or REACTOME analysis. Arrows indicated REACTOME-curated data. For validation, we used experimental gene expression data from osteoclasts and osteoblasts in combination with statistical testing. The resulting gaussian probability of interactions, i.e. FDR-adjusted p-values (q-values) were superimposed on the STRING PPIs as follows: large line width, q<0.0005; medium line width, q<0.05; small line width, q>0.05 or no q data. (B) Based on their cellular expression, the three class 2 low BMD candidate genes Rab31p, Ncald, and Arl4d were probed in the osteoclastic and osteoblastic compartments of bone turnover. Rab3ip and Ncald interacted via single intermediates with proteins degrading bone matrix and also mineral, while Arl4d, also via an intermediate, interacted with a regulator of the procollagen cleavage essential for bone matrix formation.

The finding that class 2 genes causing low BMD, such as *Arl4d*, *Ncald*, and *Rab3ip*, participated in osteoclast or osteoblast pathways, raised the possibility that they are involved in the skeletal mechanisms crucial for maintaining bone mass, particularly bone turnover. In the absence of established methods that permit rapid testing of potential bone turnover genes, without the need for animal experimentation, we devised a novel theoretical approach. As bone resorption and formation underlying bone turnover occur in a spatial and temporal fashion in the extracellular space, we exploited the known key proteins responsible for osteoclastic resorption of mineralized bone matrix and subsequent osteoblastic matrix formation and mineralization. The PPIs between these proteins were recorded and created an *in silico* bone turnover model (Fig 6A). We then introduced the three low BMD class 2 genes products Rab3ip, Ncald, and Arl4d, and found all three genes affiliated with essential extracellular bone turnover proteins in each case via a single intermediate. The model showed that during extracellular bone resorption, Rab3ip strongly interacted via Racgap1 with Mmp14 but also with Mmp9 and Atp6v0d2, suggesting an effect on both bone matrix and mineral degradation (Fig 6B). In contrast, Ncald interacted via Fgf2 only with Mmp9 and Mmp14 (Fig 6B), indicating a sole effect on matrix degradation. With respect to bone formation, Arl4d interacted via Kdelr3 with Pcolce (Fig 6B), possibly impacting bone matrix assembly. This further supported a role of Arl4d in osteoblast-mediated BMD maintenance.

Because BMD proteins and their networks are contingent on the transcription of the corresponding BMD genes, we examined whether BMD gene expression was regulated on the transcriptional level. Within the pool of the 200 BMD genes, we probed for enrichment of TFs in promoters of genes causing low compared to high BMD phenotypes upon deletion (Table 2). A total of 49 and 32 TFs were identified for low and high BMD, respectively. In addition, we examined the 200 BMD genes for TFs and found 17 and 13 TFs that caused low and high BMD phenotypes, respectively (S6 Table). Together, these data (1) demonstrated the transcriptional regulation of BMD phenotypes, (2) recognized a total of 127 TFs involved in the regulation of low and high BMD genes, and (3) discovered 74 TFs that were not reported previously to be skeletal TFs.

**Table 2:** TFs enriched in low or high BMD genes (i-CisTarget analysis).

Low BMD							High BMD					
	*TF*	*NES*	*BMD Genes*			*STF*	*TF*	*NES*	*BMD Genes*			*STF*
			*Class 1*	*Class 2*	*Total*				*Class 1*	*Class 2*	*Total*	
1	Bnc1	4.516	3	11	14	Yes	Maz	5.739	7	4	11	Yes
2	Mxi1	4.513	16	45	61	Yes	Scrt2	4.334	5	9	14	No
3	Tga2	4.427	4	9	13	No	Hif1a,Arnt	4.219	5	3	8	Yes
4	Creb3l1	4.400	10	19	29	Yes	Caco2	4.198	6	4	10	No
5	Hcfc1	4.028	7	17	34	No	Foxd1	4.016	8	6	14	Yes
6	Fbp6	3.866	2	13	15	No	Hif1a	3.861	12	14	26	Yes
7	Zfp260	3.839	2	14	16	No	Bach1	3.838	6	6	12	No
8	Polr2a	3.793	18	56	74	No	Xbp1p	3.668	4	4	8	No
9	Nac6	3.727	5	8	13	No	Madf	3.667	7	11	18	Yes
10	Foxo4	3.718	6	10	16	Yes	Nf1	3.605	6	5	11	No
11	Mads3	3.717	6	13	19	No	Bhlhe40	3.552	6	4	10	No
12	Zmiz1	3.652	7	7	14	No	Hes2,Hes5	3.487	3	9	12	Yes
13	Zfp1	3.621	5	12	17	No	Srf,Rfx3	3.407	5	7	12	Yes
14	Ascl2,ascl1	3.565	5	12	17	Yes	zf-C2H2	3.349	5	8	13	No
15	Hlf	3.505	6	9	15	No	Evx2	3.333	4	5	9	Yes
16	Zfp92	3.436	6	6	12	No	Evx1	3.319	4	5	9	Yes
17	Zbtb33	3.411	24	62	86	Yes	Zfp62	3.307	5	7	12	No
18	Hy5	3.389	6	9	15	No	Rbak	3.295	4	3	7	No
19	Ets1	3.388	7	8	15	Yes	Zfp667	3.286	7	7	14	No
20	At4g28140	3.354	4	9	13	No	Klf11	3.279	15	18	33	No
21	Jun	3.348	5	18	23	Yes	Klf10	3.270	15	18	33	Yes
22	Jund	3.330	2	9	11	Yes	Klf13	3.270	13	18	31	No
23	Gbf1	3.328	4	9	13	No	Zbtb7c	3.259	16	28	34	No
24	Cej1	3.324	1	9	10	No	Srebf1	3.232	5	6	11	Yes
25	Rap2	3.300	5	10	15	No	Bteb1	3.162	14	18	32	No
26	Yy1	3.292	5	12	17	Yes	Sall2	3.155	6	7	13	No
27	Mads26	3.287	5	13	18	No	Znf224	3.114	5	7	12	No
28	Tavrt-1	3.284	6	11	17	No	Tbx3,Hoxb13	3.051	5	3	8	Yes
29	Eomes	3.261	3	6	9	Yes	Tfeb	3.044	5	5	10	Yes
30	Mads11	3.255	8	11	19	No	Zrsr2	3.043	6	5	11	No
31	Opaque-2	3.252	9	15	24	No	Pitx1,Hoxa3	3.020	6	5	11	Yes
32	E2f6,nr3c1	3.240	6	11	17	Yes	Cbf1	3.013	7	6	13	Yes
33	Erf,nhlh1	3.230	4	7	11	Yes
34	Foxo1,elk1	3.197	3	11	14	Yes
35	Nfil3	3.171	16	42	58	Yes
36	Sin3a	3.151	7	20	27	Yes
37	Znf418	3.146	13	29	42	No
38	Fbp21	3.142	8	12	20	No
39	Foxo1,fli1	3.138	1	10	11	Yes
40	Ets1,tfap4	3.121	4	15	19	No
41	Xbp1	3.104	4	11	15	Yes
42	Zfp408	3.086	3	11	14	No
43	Mads2	3.086	5	11	16	No
44	Elf1,foxo1	3.083	3	11	14	No
45	Nr2e1	3.063	5	16	21	No
46	Myc	3.057	4	5	9	Yes
47	Cbt	3.038	3	8	11	Yes
48	Znf256	3.025	5	15	20	No
49	Creb3l2	3.002	5	5	10	Yes

Abbreviations: BMD, bone mineral density; NES, enrichment score; STF, known skeletal TF; TF, transcription factor.

## Discussion

On behalf of the IMPC, we have summarized the IMPC skeletal data. The first question this study addressed was whether screening of mutant mice at scale would identify novel genes controlling BMD. Phenotyping for BMD was performed on 3,823 mutant mouse strains. Compared to wt controls, we observed altered BMD in 200 mutant strains (Fig 2A). Analysis of this cohort led to the identification of 141 class 2 genes with a previously unknown function in controlling BMD (Table 1).

The magnitude of BMD change in response to gene deletion followed a multi-modal distribution (Fig 2C). A high percentage of the 200 BMD genes were associated with a decrease in BMD between 5% and 10%. Despite the modest decrease in BMD, we consider these genes significant, because osteoporosis is believed to be a largely polygenic disorder, determined by the effects of several genes, each with relatively modest effects on bone mass [22]. Further, we identified a number of genes producing greater than 10% decreased or increased BMD (Fig 2D and 2E), including novel class 2 genes, for example *Nhlh2* and *Dppa*. A loss of BMD greater than 20% was recorded for *Lepr*, *Bbs5*, *Cyp27b1*, and *Ghrh*, while a gain in BMD of more than 20% was only noted for *Lrrk1* (Fig 2C–2E and S1 Table). These five class 1 genes are all known key players in bone biology, and their respective effect on BMD should be considered confirmatory.

The human relevance of the presented mouse BMD genes was gauged by comparing the mouse IMPC data to human GWAS data derived from the UK Biobank. An overlap of up to 25% (S3 Table) was seen. Further, a more detailed GWAS analysis based on the GEFOS data set found that, of the 200 BMD genes, one class 1 and six class 2 genes were linked to corresponding human genes (S4 Table). Together, this showed that mouse-derived BMD data has relevance for human BMD pathologies, but it also demonstrated that the IMPC mouse data is complementary to GWAS-derived human data and has the potential for *de nov*o gene discovery. Importantly and in contrast to GWAS studies, the IMPC data is based on targeted gene disruption, thus potentially offering causal validation of candidate genes found via SNP analysis in GWAS. We anticipate that future progress in the understanding of osteoporosis genetics hinges on the synergistic application of both human GWAS and the IMPC mouse phenotyping program. At present, the IMPC BMD data is similar in size to a skeletal phenotyping program that was carried out between 2000 and 2008 in a commercial setting at Lexicon Pharmaceutical [23]. However, the IMPC program will continue to grow and thus the skeletal data presented here will expand in size and depth over time.

A gender-specific action of BMD genes has been postulated previously but led to inconclusive results. On the one hand, a recent GWAS study dedicated to the assessment of gene-by-sex interaction, did not find such an interaction for BMD [24]. On the other hand, a string of earlier studies using whole genome quantitative trait loci analyses identified chromosomal regions linked to sex-dependent regulation of BMD [25–27]. Further, studies using individual gene deletion in mice, proved that genes cause gender-restricted effects on BMD [28]. Lastly, it has been shown that the regulation of a gene can vary between the sexes [29]. In the present study, which we believe is currently the most comprehensive analysis reporting BMD changes in both male and female mutant mice, we defined 90 genes that, upon deletion, caused a change in BMD that was restricted to either female or male animals (Fig 3A–3C). Notably, in females the number of genes causing low BMD was about 4-fold greater than the number of genes producing high BMD (Fig 3A and 3B). In contrast, in males a comparable number of genes were associated with low and high BMD (Fig 3A and 3B). The reason for this distribution is currently unknown. Preliminary annotation of the 90 genes identified most frequently energy metabolism pathways (S7 Table) and was not restricted to hormonal pathways known to affect bone [30]. Together, our data further supports the hypothesis of sexual dimorphism in BMD gene regulation.

The 200 BMD genes discussed above, included 59 class 1 genes. Although these class 1 genes have a known function in bone biology, our study revealed a previously unknown function in BMD control for about half of the 59 genes (S8 Table), and we plan on further investigating these BMD genes in separate studies. Another important aspect of the class 1 gene set is that it offered an opportunity to validate the presented large-scale phenotyping approach. Comparing our BMD data to previously published reports, we found an agreement for 57 out of 59 genes or 95% (S8 Table). Given that large scale mouse screenings use a highly pre-defined analysis pipeline with little option for tailored analysis or cell biology studies, we felt an error rate of 5% supported the robustness of the presented data. A clear discrepancy between our findings and previous data was seen for two genes. First, Ndrg1, a ubiquitously expressed protein with unclear cellular function. Deletion of his gene was previously reported by Watari *et al*. on a B6 background and produced animals of short stature that exhibited neurological and skeletal abnormalities, including spinal curvature and an at least 60% increase in BMD [31]. The IMPC model reported here matched those phenotypes with the exception that we did not record spinal changes and observed a <5% decrease in BMD. These differences may result from different gene targeting strategies or skeletal analyses. For example, Watari *et al*. employed *in vitro* microCT analyses, while we used *in vivo* DXA. Further, Watari *et al*. measured volumetric BMD on the femur and we reported areal BMD across the skeleton, excluding the skull. A second gene with conflicting results was *Col1a2*. This gene codes for the collagen type I alpha 2 chain, which together with the collagen type I alpha 1 chain, form hetero-trimeric procollagen. We were surprise to measure a slightly increased BMD in *Col1a2* deficient mice. Loss of *Col1a2* in mice has been studied previously [32]. For example, in the osteogenesis imperfecta murine (oim) model, a single nucleotide deletion in the *Col1a2* gene, resulted in a normal size mRNA, but fail to secrete the protein. Bones in these mice are composed of Col1a1 chains only, forming homo-trimeric type 1 collagen. Phillips *et al*. reported reduced BMD in oim mice based on DXA measures. In line with these findings, the Amish Col1a2 mouse model exhibited reduced BMD across several genetic backgrounds [33]. Further, homo-trimers due to *COL1A2* mutations have been reported in patients and manifested in forms of osteogenesis imperfecta or Ehlers-Danlos syndrome [34]. In some patients, BMD was measured and generally found to be decreased [35–37]. Interestingly, mutations that specifically affect the C-propetide cleavage site of COL1A2 result in high BMD [38]. Taken together, previous data from mice and humans let us to expect a decreased rather than increased BMD as a result of *Col1a2* deletion in mice. Hence, for our *Col1a2* mutant in particular, the inherent limitation of large-scale phenotyping became apparent because molecular and cell biology studies are essential to verify the expected mRNA status and collagen trimer composition. The comprehensive dataset we reported in this study also included 3,623 genes that upon deletion did not show a significant BMD phenotype (S2 Table). A preliminary assessment of these genes showed that the false negative rate was about 2%, further supporting the robustness of our data (S2 Table). Within the pool of BMD negative genes, two genes illustrated principle restrictions in our approach. First, we did not detect a change in BMD upon deletion of *Daam2*. Yet, using GWAS for both BMD and bone fracture as well as mouse and *in vitro* studies, Morris *et al*. identified *Daam2* as a potential genetic factor in osteoporosis [10]. In fact, they investigated the same *Daam2* KO mouse as we did, however they relied on the OBCD pipeline for skeletal analyses. Consistent with our findings, no BMD alteration was seen, but bones of KO mice showed reduced mechanical stability. This example highlights the previously discussed limitation of sole BMD measures for the detection of osteoporosis genes [39]. Second, based on the IMPC data release 6.0, which we explored in this study, we observed no BMD phenotype after deletion of *Slc20A2*. However, the ongoing IMPC program constantly adds data on new mouse KO lines, and thus over time the pool of control mice may expand or changes to the PhenStat statistical package introduced. This can affect data output. In fact, in IMPC data release 11 the gene *Slc20A2* has been assigned a BMD phenotype. From a comparison between different IMPC data releases, we estimate that such shifts in BMD phenotype hits may occur in the order of 0.5% of genes per release. Regardless, we recommend that investigators interested in skeletal studies using IMPC mouse models (a) re-examine the mutant mice and matching wt controls using *in vivo* DXA and standard laboratory and animal group sizes, and (b) perform *in vitro* microCT at least including parameters measured under the OBCD pipeline, such as femur bone volume/tissue volume, BMC, and BMD as well as several structural measures derived from cancellous and cortical bone.

The 141 class 2 genes constitute an important stand-alone data set, as they provide experimental *in vivo* evidence justifying the molecular and cellular investigation of a comparably large number of gene products previously not known to affect BMD. In this study, however, we sought to address further questions. We asked which of the 200 BMD genes caused both BMD alterations and skeletal abnormalities. A set of 19 class 2 genes was found (Fig 4C). To assess a potentially compromised skeletal stability in mice lacking one of these genes, we initiated and executed the import of data from the OBCD program into IMPC, a step necessary for proper data analysis. Despite the currently limited overlap (around 10%) of genes in the IMPC and OBCD programs, Hbs1l could be analyzed and was found to yield an altered, reduced skeletal stability after ablation. This is an important finding because bone fragility is a hallmark of osteoporosis. However, loss of *Hbs1l*, which codes for a GTP-binding elongation factor, yields several co-phenotypes and thus further examination of tissue-specific functions of Hbs1l is needed. Noteworthy, a brief report previously described the occurrence of a *HBS1L* mutation in humans [40], and the IMPC knockout mouse model is currently used to further investigate Hbsl1 loss [41]. Independent of the OBCD program, we considered more general criteria useful for the identification of promising new BMD genes. Arguably, we used a limited weight loss and low number of co-phenotypes as indicators for a direct skeletal action of a gene. The six genes, *Arl4d*, *Sdsl*, *Bbx*, *Fam160a1*, *Pld5*, and *Tnfaip1*, with both limited body weight alteration and low pleiotrophy.

Another question we asked was whether evidence existed for functions of BMD genes in osteoclasts or osteoblasts. Our experimental approach was to use available gene expression data for assignment of the 200 BMD genes to osteoclasts and osteoblasts. For high confidence, curated data acquired during bone cell differentiation was used. We utilized this data for two purposes. First, we simply probed for cellular expression (Fig 5). Here, we noted that several class 1 osteoclast genes, like *Ckb*, *Ndrg1* and *Sat1b*, were also detected in osteoblasts (Fig 5). Because of a lack of verification of their biological function in osteoblasts, we did not further consider these genes. Second, gene expression data was included in the more sophisticated calculations of protein interaction probabilities (Fig 6). Concerning both uses, we emphasize that expression data depends on biological factors, including cell type and time-point investigated, and may only present a snapshot of a biological system. With these limitations in mind, we assigned the BMD genes to osteoclasts and osteoblasts and surveyed for protein-protein interactions using STRING (Fig 5). Because the action of genes occurs on the protein level, testing for protein-protein interactions has merit. STRING also provides an opportunity to investigate gene products lacking extensive prior characterization. Despite these advantages, our STRING-based analyses had two principle limitations. Firstly, the interactions shown in the networks comprised a range of interaction modes, which were not always experimentally verified. Secondly, in addition to the BMD genes identified in this study, the networks incorporated gene products that do not have an experimentally confirmed role in bone cells. Regardless, several genes with a known function in bone cells (class 1), such as *Ctnnb1* [42, 43], were detected and validated our approach.

With respect to proteins previously not noted to have a function in bone biology (class 2), we report several intriguing findings. In osteoclasts, STRING mapped the protein phosphatase Pptc7 which has a clear function in CoQ10 biosynthesis [44], a biochemical pathway with an established role in osteoclasts [45]. However, Pptc7 is also localized in the plasma membrane (PM) and STRING predicted an interaction of Pptc7 with the guanine nucleotide exchange factor Rab3ip via Exoc2, a component of the exocyst complex. Rab3ip is a class 2 protein with a known function in vesicle transport and together with Exoc2 may mediate transport to the plasma membrane and thus may participate in the vesicle transport of Pptc7 to the PM [46]. STRING analysis also demonstrated an interaction of Rab3ip with the ADP-ribosyltransferase Arf4. Like Rab3ip, the Arf-family of small GTPase, including Arf4, functions in vesicle trafficking [47]. Moreover, an interaction of Arf4 with the class 2 gene *Ncald* was found. Ncald is a regulator of G-protein receptor signaling and has a function in calthrin-coated vesicle transport [48]. Importantly, data has suggested that clathrin-coated vesicle transport plays a role in osteoclasts [49]. Together, the class 2 genes *Rab3ip* and *Ncald*, which both cause low BMD upon deletion (Table 1), are likely to participate in vesicle trafficking in osteoclasts, supporting the emerging role of membrane trafficking in bone biology and pathology [49]. Regarding osteoblasts, STRING mapped the Arf-like (Arl)4d protein, which upon deletion caused both low BMD (Table 1) and skeletal abnormalities. i.e, abnormal rib morphology (Fig 4C). It further proposed an interaction of Arl4d with the KDEL endoplasmic reticulum protein retention receptor 3 (Kdelr3), and also a Kdelr3 interaction with Col1A2. The latter is a major component of the bone matrix and is synthesized, heterodimerized with Col1A1, and secreted as procollagen. The underlying secretory pathways are not fully elucidated but appears to include secretory vesicles/granules tailored to the rigid and bulky procollagen load [50]. The interaction of Kdelr3 with Col2a1 is likely based on the prominent function of the KDEL receptors in cis-Golgi sorting and recycling of KDEL-tagged proteins, such as the collagen-specific chaperon Hsp47 [51]. The interaction of KDEL receptors with Arl4d, however, might be different from their interaction with collagen. Data has shown that KDEL receptors control the recruitment of cytosolic GTPase activating proteins (GAPs) to plasma membrane (PM)-bound Arfs [52]. As GTPases, Arfs are not only regulated by GAPs, but also guanine nucleotide exchange factors (GEFs), necessitating GEF recruitment to the PM. Studies have reported localization of Arl4d to the PM where it functions to recruit cytohesins, including the GEF ARNO. This GEF controls Arf6, which is a known regulator of endocytotic traffic, but also exocytotic granule transport [53]. Therefore, the study presented here led to the working hypothesis that in osteoblasts loss of Arl4D compromises the exocytotic vesicle transport of bone ECM components. The above findings were supported by interpretation of the *in silico* bone turnover model (detailed in S1 Text), which let us to speculate that (a) Rab1ip acts through Rab8b and Rho signaling, (b) Ncald, in the presence of FGF-2, engages with guanylyl cyclase-B, and (c) Arl4d is involved in the exocytotic vesicle-based secretion of Pcolce. We emphasize that the *in silico* bone turnover model at present has a limited and biased selection of genes and needs experimental validation. It may, however, prove useful for other large-scale genetic studies on bone for three reasons. First, it is compatible with gene sets from a variety of sources and species. Second, it can be easily recreated by other laboratories at no cost. Lastly, the model is expandable to include additional genes and tailorable to specific research questions.

Taken together, this study (1) identified and characterized BMD genes, including 141 class 2 genes that lacked prior association with bone biology, (2) prioritized the class 2 genes based on magnitude of BMD change, human relevance, or pleiotropy, (3) offered clues on pathways, such as vesicle transport, governing bone cell biology in BMD pathologies, and (4) underscored the potential role of the genes, such as *Arl4d*, *Ncald*, and *Rab3ip*, in low BMD pathologies, including osteoporosis.

## Materials and methods

### Ethics statement

All animal work described in this study was carried under the auspice of approved animal protocols (Baylor College of Medicine, #AN-5896; German Mouse Clinic Helmholtz Zentrum München, #144–10, 15–168; Institut Clinique de la Souris Mouse Clinical Institute, #4789-2016040511578546v2; Medical Research Council Harwell, #30/3384; Nanjing University, #NRCMM9; Rikagaku Kenkyūjo Tsukuba Institute, #Exp11-011, 12–011, 13–011, 14–009, 14–017, 15–009, 16–008; The Centre for Phenogenomics, #0153, 0275, 0277, 0279; The Jackson Laboratory, #11005, UC Davis, #20863).

### Generation of mutant mouse strains

The IMPC systematically phenotypes mice that are homozygous for a single-gene knockout or heterozygous when homozygotes are lethal or sub-viable. IMPC members and non-members are free to nominate genes for deletion, and mouse production was coordinated by iMits (Fig 1A). The gene-targeting strategies that are used can be accessed via http://www.mousephenotype.org/about-ikmc/targeting-strategies. For every IMPC deleted gene, specifics on the targeting strategy can be obtained through a gene search on the IMPC website (http://www.mousephenotype.org) and use of the “Order Mouse and ES Cells” tab. In the case of single copy genes, hemizygous knockout mice are studied. IMPC mouse models are available to the research community via the IMPC website.

### Mouse phenotyping

Data was derived from postnatal mice that were phenotyped under the adult and embryonic phenotype pipeline (https://www.mousephenotype.org/impress). Briefly, this pipeline conducted 16 tests, each with a set of phenotyping procedures. Phenotyping recorded appearance, behavior, or organ function across a spectrum of organs and tissues. All IMPC phenotyping data is shared with the public through the IMPC website. Experimental procedures are detailed also under IMPRESS (https://www.mousephenotype.org/impress). Although we mined the entire phenotyping data when investigating co-phenotypes, our study primarily investigated the X-ray phenotyping data obtained in postnatal week 14. The skeletal X-ray exams encompass radiographs and DXA-based measures of BA and BMC. Radiography and DXA are mandatory and voluntary IMPC measures, respectively. Specifically, for the data reported in this study, they were carried out using the UltraFocus DXA (Faxitron Bioptics LLC), the Lunar Piximus II (GE Medical Systems), or the pDexa sabre (Norland Stratec) instruments. For DXA, a minimum of 7 male and 7 female mutants were phenotyped, giving a minimum of 14 mice per line, while for radiography phenotyping was performed on a minimum of 4 male and 4 female mutants. In all experiments, mutants were matched with wild-type (wt) animals, with matching genetic background. Mutant and wt mice were phenotyped in the same manner based on the IMPReSS protocol, and detailed experiment characteristics captured in the procedure metadata. BMD was calculated from the measures of BA and BMC. For data analysis, mutants were matched with wt animals from the same center that used the same metadata.

### IMPC data analysis

The IMPC uses a bespoke statistical package, implemented in R, called PhenStat, which was developed for identification of abnormal phenotypes from high-throughput pipelines [54]. The bone mineral data was analyzed by PhenStat using linear, mixed models, which take factors such as sex and body weight into account. S1 Table details the number of wt control mice analyzed for each of the 200 BMD genes. PhenStat identifies phenotype hits by sex to enable identification of sex-specific phenotypes which have been previously shown to be relevant in a large proportion of IMPC lines across many procedures [17].

Phenotype hits were identified by searching the IMPC database (version 6.0, released November 10^th^, 2017, data access: https://www.mousephenotype.org/data/previous-releases/6.0) for Mammalian Phenotype (MP) terms as listed in IMPReSS. For bone mineral density, that includes the terms Increased Bone Mineral Density (MP:0000062) and Decreased Bone Mineral Density (MP:0000063). For those lines found to have BMD phenotypes, radiography-related MP terms, as listed in the IMPReSS X-ray procedure, were also queried to identify any skeletal phenotypes (www.mousephenotype.org/impress/procedureontologies/91/7).

### Gene classification and assessment of sexual dimorphism

To learn more about the BMD phenotype genes and to classify genes as known or unknown to be associated with bone PubMed was searched using the gene names along with the search terms “osteoblast”, “osteoclast”, “osteocyte”, “osteogenic”, “skeletal” and “bone” NOT “bone marrow”. These broad terms were selected empirically to cover a wide range of bone biology. We also repeated the same searches, but with the inclusion of “mouse” and “human”. Our search did not encompass other skeletal tissues such as mesenchymal stem cells, cartilage, dental tissues, or malignancies. The literature search was closed on June 16, 2018.

Genes controlling BMD in a sex-biased fashion were retrieved from the 200 BMD phenotype genes. MGI and GeneCards were used to identify the chromosomal localization of genes in mice and humans, respectively. To identify genes described previously in the context of sexual dimorphism we used PubMed in combination with the search terms “sexual dimorphism”, “sexual”, “sex”, “male AND female”.

### Mapping genes to gene ontology terms

GO terms for the genes of interest were extracted from the MGI database (www.informatics.jax.org), which contains information on genes and their annotated phenotypes. In order to avoid circular findings, as the MGI database includes IMPC phenotype data, IMPC entries were removed using MGI’s internal filters. We analyzed terms of the “molecular function” and “biological processes” domains as they provide information on the biological activity of genes of interest. To identify genes involved in skeletal function based on gene ontology term annotation, we selected all gene ontologies that included the terms “bone”, “skeletal”, “ossification”, “osteoclast” and “osteoblast”. MGI’s Gene eXpression Database www.informatics.jax.org/expression.shtml) was also searched to determine the known expression profiles of the BMD phenotype genes.

### Genome-wide association studies (GWAS)

Orthologous/paralogous genes of mouse metabolism genes mapping to the human genome were used for analysis. GWAS analyses on human BMD were based on data acquired in the UK Biobank (www.ukbiobank.ac.uk) [9, 10, 55] and GEFOS (www.gefos.org) [8] studies. We used the Ensembl BioMart online tool to adjust all genomic data to the latest Assembly (GRCh38/hg38). The UCSC genome browser was used to analyze genes in detail. We searched for SNPs in a ±2 kb region in the SNiPA database. For each SNP occurring in or around genes, we evaluated the extent of sharing based on the three bone density phenotypes recorded by the GEFOS consortium, and used metadata without any link to individual IDs or data only. We used cross phenotype meta-analysis (CPMA), which detects association of a SNP to multiple, but not necessarily all, phenotypes [56]. The CPMA analysis applies the likelihood ratio test that measures the likelihood of the null hypothesis (i.e., that the significant SNP is uniformly distributed across consortiums) over the alternative hypothesis.

### OBCD data

The OBCD (www.boneandcartilage.com) is a project performing bone and cartilage-related phenotyping on knockout mouse models. The bone-based procedures performed include three-point bend and vertebra compression, for which they currently have data for a total of 410 lines. These procedures were carried out in female animals only. In male animals, rapid-throughput joint phenotyping was performed instead. Analysis of the female OBCD bone data set was based on a tight reference data obtained from a population of >300 strain-matched female wt mice, giving it the power to detect differences between controls and small cohorts (two female animals per line) of mutant mice [5, 9, 57]. To identify lines with data differing from the wt, those with measurements greater than two standard deviations from the wt mean were considered significant.

### Gene expression analysis

The NCBI Gene Expression Omnibus was used to analyze gene expression in osteoblasts and osteoclasts. To assure robustness of the expression data, the analysis was restricted to the curated data available under https://www.ncbi.nlm.nih.gov/sites/GDSbrowser/, and only data on mouse-derived cells was considered. To identify genes expressed in osteoclasts or osteoblasts, we assessed expression data acquired during differentiation of these cells. Genes significantly (p<0.05) increased during differentiation were considered. For osteoclastogenesis, data sets GSE43811 and GSE57468 (series GDS5398 and GDS5422, respectively) were used. GSE43811 compared osteoclast differentiation at 48 hrs between osteoclast precursor cells (GSM 1071626, 1071627, 1071628) and control cells (GSM 1071629, 1071630, 1071631). In Additionally, we selected GSE57468, which compared differentiation of primary osteoclast precursors between days 0 (GSM1383260, 1383262) and 3 (GSM1383261, 1383263). For osteoblastogenesis, the data set GSE2332 (series GDS1631) was selected because it permitted for the study of highly enriched primary pre-osteoblasts. Differentiation days 7 (GSM43188, 43202, 43209) and 17 (GSM43195, 43216, 43225) were included. All data was analyzed using the dedicated NCBI Geo2R suite (https://www.ncbi.nlm.nih.gov/geo/geo2r/).

### Examination of protein-protein interaction (PPI) networks and the *in silico* bone turnover model

All PPI networks were built in STRING DB (https://string-db.org/). The proteins coded by the 200 BMD genes served as input or “seeds”. Our analysis utilized minimum networks generated by NetworkAnalyst. Based on the “String04 all sources” mode, up to 86% of the seeds were annotated (172 BMD proteins) with at least one PPI. Using these networks we calculated different centrality indexes using the cytoHubba app in Cytoscape to identify hubs central for maintaining the communication flow on the different parts of the network. To assess the significance of the network, it was then tested for the likelihood of observing a network with a the same degree of connectivity by comparing the reciprocity of the network versus 100,000 simulated networks of the same degree using the statnet and sna R packages (https://cran.r-project.org/web/packages/statnet/index.html, https://cran.r-project.org/web/packages/sna/index.html). For combined analysis of BMD proteins and TFs, the yfiles layouts were employed in Cytoscape. The radial layout algorithm was chosen. It portraits interconnected ring and star topologies. This algorithm produces layouts that emphasize sub-groups and tree structures within a network. It creates node partitions by analyzing the connectivity structure of the network and arranges the partitions as separate circles or disks. To develop networks representing an *in silico* bone turnover model, we used clinical markers of bone turnover as a guide for the identification of the most established proteins participating in bone turnover [58, 59]. Our final selection of bone turnover genes was supported by published data [60–62]. To experimentally validate the *in silico* model, we used gene expression data together with the R package GeneNet 1.2.13 (http://www.strimmerlab.org/software/genenet/). Based on gene expression data this package enabled the statistical assessment of the reliability of a given network (nodes and edges) and to compute q-values and posterior probabilities (= 1-local FDR) for each potential edge connecting in a pairwise manner the nodes of the network (S9 Table). We captured the bone resorption-formation sequence characteristic for bone turnover with the following expression data sets (1) GSE43811 (GSM 1071626, 1071627, 1071628 and GSM 1071629, 1071630, 1071631), (2) GSE57468 (GSM1383261, 1383263 and GSM1383260, 1383262), (3) GSE2332 (GSM 43191, 43212, 43219 and GSM 43184, 43205, 431198), (4) GSE37676 (GSM 25432, 25433, 25434 and GSM 25429, 25430, 25431), (5) GSE2332 (GSM43195, 43216, 43225 and GSM43188, 43202, 43209). The normalized log2FC expression values (GSE158151) from these datasets were used as input files for GeneNet [63].

### Promoter analysis

To identify TFs that may regulate genes implicated in bone phenotypes, we surveyed for the bone transcriptional targets, which contain common TF binding sites in their cis-regulatory control elements. Using i-CisTarget and two datasets as input; genes with increased bone mineral density and genes with decreased bone mineral density, we detected motifs within 10Kb around the transcription start site. Every gene was scanned for motifs using a library of approx. 10k PWMs and 1120 Chip-seq tracks, each motif is scored with an algorithm called Cluster-buster and results in a subset of genes that are predicted as direct targets.

## Supporting information

S1 Fig Change in BMD from baseline for lines where direction of change differed by sex. (PDF)

S1 Table Overview of all 200 BMD genes with significant BMD differences from baseline, including sex, percentage change and difference in weight. (XLSX)

S2 Table Genes with BMD data but no BMD phenotype. (XLSX)

S3 Table *Overlap of the 200 BMD genes with GWAS genes based on the UK Biobank release*. The distance dependency of the overlapping genes is shown in green, yellow, and blue, data sets. [9, 10, 55]
(XLSX)

S4 Table Sub-set of genes with human BMD phenotype. (PDF)

S5 Table Top-level skeletal co-phenotypes identified for the 200 BMD genes. (XLSX)

S6 Table TFs causing mouse BMD phenotypes upon ablation. (PDF)

S7 Table KEGG Pathway analysis of the 90 genes causing sex-specific changes in BMD. Data is organized in four groups by sex and direction of BMD change.
(XLSX)

S8 Table Validation of class 1 gene BMD data against previously published findings. (XLSX)

S9 Table Probability calculations of protein-protein interactions in the in silico model of bone turnover. (XLSX)

S1 Text Discussion of the in silico bone turnover model. (DOCX)
