# Supplementary figures and images for: Mouse mutant phenotyping at scale reveals novel genes controlling bone mineral density
Source: PLoS Genet. 2020 Dec 28;16(12):e1009190. doi: 10.1371/journal.pgen.1009190 (PMC7822523; doi:10.1371/journal.pgen.1009190)

Change in BMD from baseline for lines where direction of change differs by sex

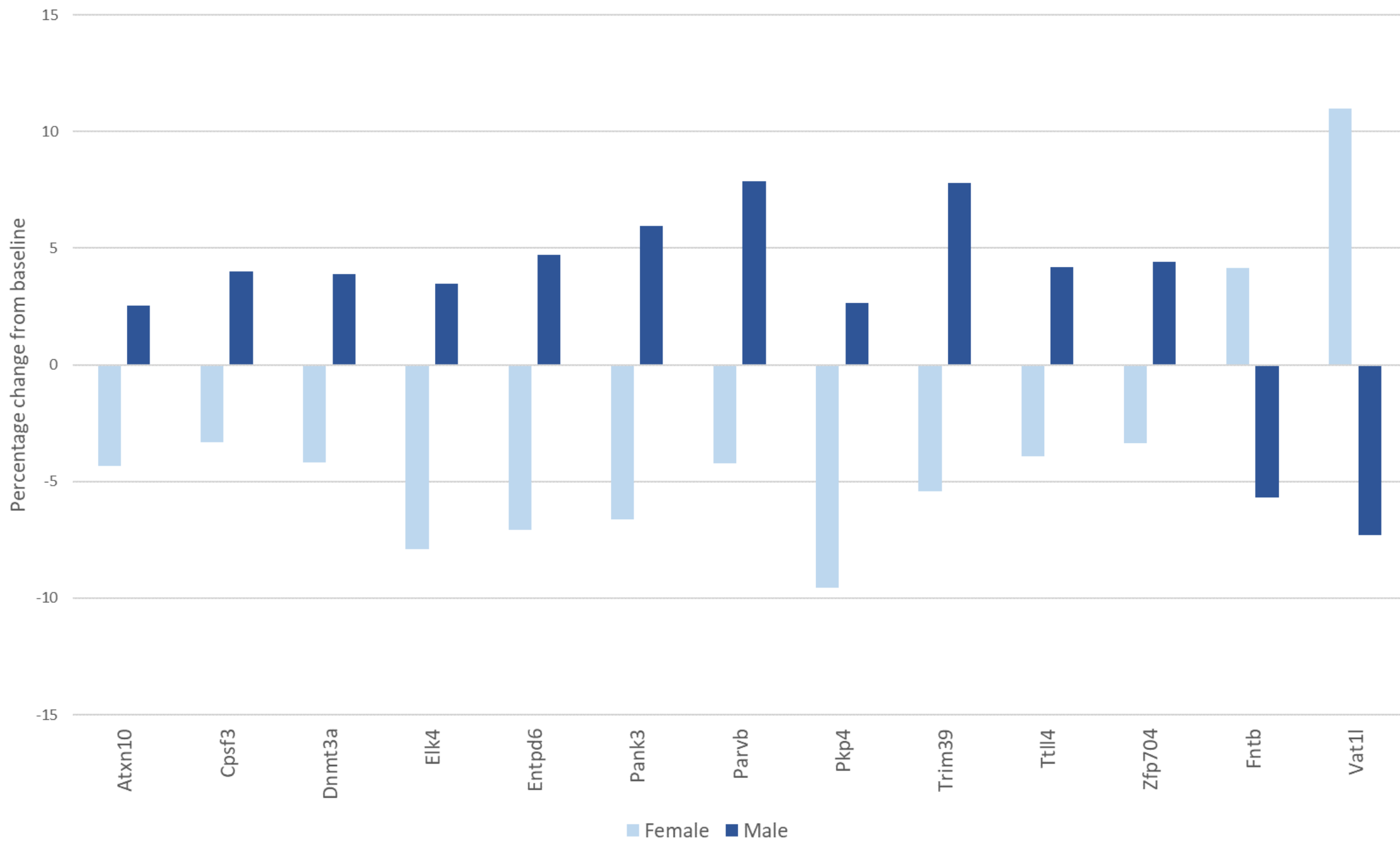

Supplement: S1 Fig — (PDF) [file pgen.1009190.s001.pdf]
