# Supplementary material for: Mouse mutant phenotyping at scale reveals novel genes controlling bone mineral density
Source: PLoS Genet. 2020 Dec 28;16(12):e1009190. doi: 10.1371/journal.pgen.1009190 (PMC7822523; doi:10.1371/journal.pgen.1009190)
Supplement: S4 Table — (PDF) [file pgen.1009190.s005.pdf]

Table: Sub-set of genes with human BMD phenotype

Gene	Chromosome	n SNPs	CPMA p-value	Top 3 SNPs	GWAS p-value	Trait	Trait direction
TTC28	22	5645	5.12E-66	rs187658153	4.50E-05	LS BMD	Decrease
				rs138288009	6.74E-05	LS BMD	Decrease
				rs146654446	9.23E-05	LS BMD	Decrease
ACSF2	17	365	1.38E-48	rs139544900	3.18E-05	LS BMD	Decrease
				rs72834156	3.98E-05	LS BMD	Decrease
				rs1870582	5.42E-05	LS BMD	Decrease
PKP4	2	2306	1.00E-01	rs115296319	4.28E-04	LS BMD	Decrease
				rs79352338	4.65E-04	LS BMD	Decrease
				rs79139823	4.83E-04	LS BMD	Decrease
MACROD2	20	21470	1.01E-99	rs73100693	9.80E-07	FN BMD	Increase
				rs73100690	1.01E-06	FN BMD	Increase
				rs365824	1.99E-06	FN BMD	Decrease
PHF19	9	256	9.88E-85	rs10760122	1.05E-05	LS BMD	Decrease
				rs881375	1.33E-05	LS BMD	Decrease
				rs1953126	1.57E-05	LS BMD	Decrease
KIAA0825	5	2429	2.10E-07	rs113118112	1.09E-05	FN BMD	Increase
				rs112217406	2.27E-05	FN BMD	Increase
				rs112268722	3.26E-05	FN BMD	Increase
AP4E1	15	782	1.44E-72	rs28642856	2.50E-04	FA BMD	Increase
				rs12899397	2.81E-04	FA BMD	Increase
				rs11854729	3.56E-04	FA BMD	Increase

Abbreviations: CPMA, cross phenotype meta-analysis; FA, fore arm BMD; FN, femoral neck; GWAS, genome-wide association study; LS, lumbar spine; SNP, single-nucleotide polymorphism.
