# Supplementary material for: Mouse mutant phenotyping at scale reveals novel genes controlling bone mineral density
Source: PLoS Genet. 2020 Dec 28;16(12):e1009190. doi: 10.1371/journal.pgen.1009190 (PMC7822523; doi:10.1371/journal.pgen.1009190)
Supplement: S6 Table — (PDF) [file pgen.1009190.s007.pdf]

Table: TFs causing mouse BMD phenotypes upon ablation

	Low BMD		High BMD	
	TF	*STF*	*TF*	*STF*
1	Adnp2	No	Bhlhe40	Yes
2	Bach2	No	Chd9	Yes
3	Bbx	No	Ctnnb1	Yes
4	Brpf1	Yes	*Dnmt3a*	Yes
5	*Dnmt3a*	Yes	*Elk4*	No
6	*Elk4*	No	Pbx3	No
7	Foxo3	Yes	Phf19	No
8	Gtf2a1	No	Rsf1	No
9	Hdac8	Yes	Satb1	Yes
10	Kmt2a	Yes	Setd1a	Yes
11	Nhlh2	No	Tcf4	Yes
12	Pdx1	No	Thra	Yes
13	Prdm14	No	*Zfp704*	No
14	Rbpj	Yes		
15	Tox	No		
16	Trip13	No		
17	*Zfp704*	No		

Abbreviations: BMD, bone mineral density; STF, known skeletal TF; TF, transcription factor. Italics indicate sex-dependent low or high BMD.
