# Supplementary material for: Mouse mutant phenotyping at scale reveals novel genes controlling bone mineral density
Source: PLoS Genet. 2020 Dec 28;16(12):e1009190. doi: 10.1371/journal.pgen.1009190 (PMC7822523; doi:10.1371/journal.pgen.1009190)
Supplement: S1 Text — (DOCX) [file pgen.1009190.s011.docx]

To permit at least *in silico* assessment of a potential role of class 2 candidate genes in bone turnover, the essential skeletal mechanism affected in many metabolic bone diseases including osteoporosis, we devised an innovative *in silico* bone turnover model (Fig. 6) and tested three class 2 candidate genes for their potential participation in bone turnover. All three genes were found to be involved in bone turnover and clues about their mechanistic role emerged. Bone turnover is initiated by osteoclastic resorption and our analysis indicated that the class 2 genes Rab3ip and Ncald participate in this process. Rab3ip demonstrated a strong interaction with genes responsible for bone matrix resorption. Mechanistically, Rab3ip converts inactive GDP-bound Rab proteins into their active GTP-bound form. Rab3ip has a preference for the small GTPase Rab8 (1), and at least Rab8b is expressed in osteoclasts (2). Several other Rab proteins function in osteoclasts, chiefly directing formation and function of the ruffled boarder of resorbing osteoclasts (3). The *in silico* bone turnover model suggested that Rab3ip functions through the GTPase-activating protein Racgap1, which is a negative regulator of Rho-signaling. Rho plays an important role in osteoclast polarity, and block of Rho has been shown to stimulate the osteoclast spreading necessary for the formation of resorptive osteoclasts (3). Hence, there is convincing evidence for further investigation of the function of Rab3ip in low BMD disease. The *in silico* bone turnover model further revealed that Ncald acts on MMP-9 and MMP-14-mediated matrix resorption via FGF-2. The mechanism of this interaction is currently unknown. However, FGF-2 is an established regulator of osteoclastic resorption (4). In addition, it has been shown to be a negative regulator of guanylyl cyclase-B (GC-B) (5), a receptor for C-type natriuretic peptide (CNP), and signaling through CNP/GC-B has a profound impact on bone growth (6). Moreover, Ncald has been demonstrated to control signaling through GC-D (7), making it reasonable to propose further studies on a potential effect of Ncald on GC-B in the presence of FGF-2. Moving from osteoclastic bone resorption to osteoblastic bone formation, the *in silico* bone turnover model reiterated the Arl4d interaction with KDEL receptors as shown in Fig. 4 and discussed above. In the in silico model, however, the interaction extended to Pcolce. We therefore speculate that Arl4d may be involved in the exocytotic vesicle-based secretion of Pcolce. As an enhancer of extracellular procollagen C-proteinase activities, including Bmp1, Pcolce is secreted from cells, but to date little is known about the exocytotic pathways of Pcolce and BMP1.

1. Hattula K, Furuhjelm J, Arffman A, Peranen J. A Rab8-specific GDP/GTP exchange factor is involved in actin remodeling and polarized membrane transport. Mol Biol Cell. 2002;13(9):3268-80.

2. Hirvonen MJ, Mulari MT, Buki KG, Vihko P, Harkonen PL, Vaananen HK. Rab13 is upregulated during osteoclast differentiation and associates with small vesicles revealing polarized distribution in resorbing cells. J Histochem Cytochem. 2012;60(7):537-49.

3. Weivoda MM, Oursler MJ. The Roles of Small GTPases in Osteoclast Biology. Orthop Muscular Syst. 2014;3.

4. Chikazu D, Hakeda Y, Ogata N, Nemoto K, Itabashi A, Takato T, et al. Fibroblast growth factor (FGF)-2 directly stimulates mature osteoclast function through activation of FGF receptor 1 and p42/p44 MAP kinase. The Journal of biological chemistry. 2000;275(40):31444-50.

5. Robinson JW, Egbert JR, Davydova J, Schmidt H, Jaffe LA, Potter LR. Dephosphorylation is the mechanism of fibroblast growth factor inhibition of guanylyl cyclase-B. Cell Signal. 2017;40:222-9.

6. Nakao K, Osawa K, Yasoda A, Yamanaka S, Fujii T, Kondo E, et al. The Local CNP/GC-B system in growth plate is responsible for physiological endochondral bone growth. Sci Rep. 2015;5:10554.

7. Venkataraman V, Duda T, Ravichandran S, Sharma RK. Neurocalcin delta modulation of ROS-GC1, a new model of Ca(2+) signaling. Biochemistry. 2008;47(25):6590-601.
